# Modulation of tumor plasticity by senescent cells: Deciphering basic mechanisms and survival pathways to unravel therapeutic options

**DOI:** 10.1590/1678-4685-GMB-2023-0311

**Published:** 2024-05-27

**Authors:** Andrew Oliveira Silva, Thais Cardoso Bitencourt, Jose Eduardo Vargas, Lucas Rosa Fraga, Eduardo Filippi-Chiela

**Affiliations:** 1 Faculdade Estácio, Porto Alegre, RS, Brazil.; 2 Centro de Pesquisa Experimental, Hospital de Clínicas de Porto Alegre, Porto Alegre, RS, Brazil.; 3 Universidade Federal do Rio Grande do Sul, Programa de Pós-Graduação em Biologia Celular e Molecular, Porto Alegre, RS, Brazil.; 4 Universidade Federal do Paraná, Departamento de Biologia Celular, Curitiba, PR, Brazil.; 5 Universidade Federal do Rio Grande do Sul, Departamento de Ciências Morfológicas, Porto Alegre, RS, Brazil.; 6 Universidade Federal do Rio Grande do Sul, Programa de Pós-Graduação em Medicina: Ciências Médicas, Porto Alegre, RS, Brazil.; 7 Universidade Federal do Rio Grande do Sul, Centro de Biotecnologia, Porto Alegre, RS, Brazil.

**Keywords:** Aging, cancer, tumor microenvironment, plasticity, senotherapy

## Abstract

Senescence is a cellular state in which the cell loses its proliferative capacity, often irreversibly. Physiologically, it occurs due to a limited capacity of cell division associated with telomere shortening, the so-called replicative senescence. It can also be induced early due to DNA damage, oncogenic activation, oxidative stress, or damage to other cellular components (collectively named induced senescence). Tumor cells acquire the ability to bypass replicative senescence, thus ensuring the replicative immortality, a hallmark of cancer. Many anti-cancer therapies, however, can lead tumor cells to induced senescence. Initially, this response leads to a slowdown in tumor growth. However, the longstanding accumulation of senescent cells (SnCs) in tumors can promote neoplastic progression due to the enrichment of numerous molecules and extracellular vesicles that constitutes the senescence-associated secretory phenotype (SASP). Among other effects, SASP can potentiate or unlock the tumor plasticity and phenotypic transitions, another hallmark of cancer. This review discusses how SnCs can fuel mechanisms that underlie cancer plasticity, like cell differentiation, stemness, reprogramming, and epithelial-mesenchymal transition. We also discuss the main molecular mechanisms that make SnCs resistant to cell death, and potential strategies to target SnCs. At the end, we raise open questions and clinically relevant perspectives in the field.

## Key concepts about cellular senescence

Senescence is a cellular state of loss of proliferative capacity due to multiple cell divisions or exposure to stresses. Telomere erosion due to the inefficiency of the DNA replication machinery at the ends of chromosomes results in blocking cell proliferation, a phenomenon known as replicative senescence. Physiologically, this mechanism helps to prevent mitotically aged cells, which potentially carry DNA changes, from transmitting genetic alterations to daughter cells. In addition to telomere shortening, stresses such as DNA damage, oncogenes activation, loss of tumor suppressor genes, or damage in cellular components like mitochondria or cytoskeleton can also induce a cell to senesce, the so-called induced (or premature) senescence ( Di Micco et al., 2021). Although a few references using genetic silencing of senescence effectors show that these strategies can possibly revert the phenotype ( Beauséjour et al., 2003; Afifi et al., 2023), *in vitro* and *in vivo* data support its irreversible nature ( Gorgoulis et al., 2019; Afifi *et al.,* 2023). It contrasts with quiescence, characterized by its reversibility and the reacquisition of responsiveness to growth factors ( Beauséjour *et al.,* 2003; Blagosklonny, 2011). Senescent cells (SnCs) exhibit distinct morphological features, including enlarged and flattened cell shape, increased cytoplasmic granularity, and altered nuclear structure including chromatin changes with the emergence of heterochromatin foci. Molecular characteristics include the upregulation of cell cycle inhibitors (e.g., p16^INK4/Arf^ and p21^CIP1^, hereafter named only as p16 and p21, respectively), DNA damage response activation (in most cases), increased activity of senescence-associated beta-galactosidase (SA-β-gal), and a secretory program called senescence-associated secretory phenotype or (SASP), consisting of soluble molecules and extracellular vesicles ( Hernandez-Segura et al., 2018; Gorgoulis *et al.,* 2019).

Although non-proliferative, SnCs are metabolically active, especially considering their secretory capacity, including the release of growth factors, cytokines, chemokines, matrix metalloproteinases, and other molecules. Thus, on the one hand, undergoing senescence helps to prevent the formation of neoplasms by avoiding the replication of cells carrying damages of different natures. On the other hand, the accumulation of SnCs in formed tumors, a feature recently included as a new hallmark of cancer ( Hanahan, 2022), could positively contribute to tumor progression.

SnCs can modulate mechanisms in neighbor cells that are not senescent. Among these mechanisms is cellular plasticity, another feature recently listed as a typical feature of cancer. This review discusses the main findings about the impact of SnCs on tumor plasticity and phenotypic transition processes, like epithelial-to-mesenchymal transition (EMT), cancer cell stemness, differentiation, and reprogramming. We also raised the main signaling pathways SnCs use to survive, which ultimately allows their maintenance in the tumor, enabling them to play their pro-tumor role while revealing potential targets to sensitize SnCs to die. At the end, we discuss critical open questions and challenges in the field of senescence, which has become one of the points of most significant translational potential in cancer biology. 

It is worth noting that, initially, studies were focused on deciphering senescence features in normal senescent cells until discovering that cancer cells can also undergo this cell fate as well, introducing the new concept of senescent cancer cells (SnCCs - i.e., cancer cells undergoing cellular senescence mainly by oncogene activation or induced by therapies). However, other cell types from the tumor microenvironment (TME) can also undergo senescence, such as stromal cells ( Guillon et al., 2019; Pardella et al., 2022; Ye et al., 2023), cancer-associated fibroblasts (CAFs) ( Higashiguchi et al., 2023), immune cells ( Bruni et al., 2019), and endothelial cells ( Abdelgawad et al., 2022; Bloom et al., 2023), highlighting the contribution of both tumor and non-tumor cells to the population of senescent cells in the TME. Since several essential senescent cell characteristics are shared by both cell types, in this review, we use ‘SnCs’ for general senescent cell behaviors and phenotypes, and ‘SnCCs’ to refer exclusively to cancer senescent cells.

## Senescence in cancer: allies turned adversaries

SnCs are found in all major human organs. Once senescent, the cell loses its proliferative capacity, becoming unresponsive to growth factors ( Hinds and Pietruska, 2017). Thus, at first, the senescent barrier acts as an endogenous antitumor mechanism, blocking the proliferative capacity of transformed cells ( Vargas et al., 2012), a premise that is reinforced by the abundance of SnCs in many benign tumors ( Collado and Serrano, 2010). However, human cells can undergo genetic or epigenetic changes that prevent or attenuate this anti-proliferative response, favoring an immortalized cellular phenotype. In this process, “immortal” tumor subclones successfully evade the senescent barrier, enabling tumor progression and the acquisition of malignant phenotype. ( Collado and Serrano, 2010). To acquire this feature, tumor cells must overcome the Hayflick limit, which denotes the finite number of divisions human cells can undergo, attributed to the shortening of telomeres ( Shay and Wright, 2000). This unlimited proliferative capacity is acquired primarily through negative modulation or loss of the TP53-p21 pathway or CDKN2A loci, which encodes the CDK inhibitors p16 and p14^Arf^. ( Takeuchi et al., 2010; Terzian et al., 2010; Hernandez-Segura et al., 2018). This molecular evasion of senescence was already described at different stages of aggressiveness of skin (melanoma), lung, colon, and breast tumors, among others ( Meeker et al., 2004; Bennecke et al., 2010). 

**Figure 1 -:**
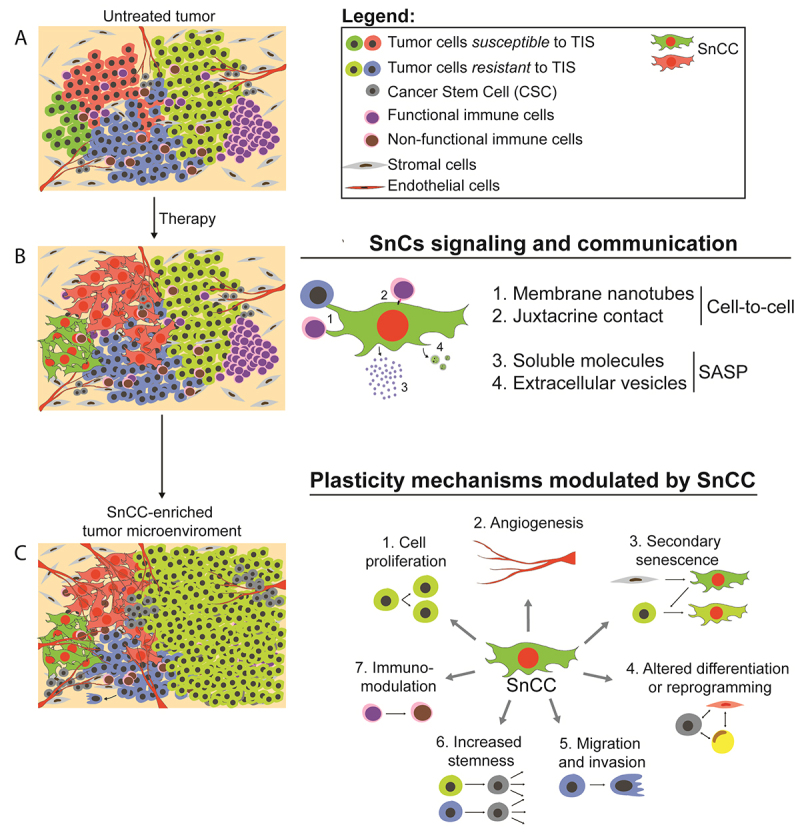
Tumor microenvironment (TME) modulation by Senescent Cells (SnCs). (A) Tumor heterogeneity is represented by tumor cell subpopulations and other cell subtypes found in the TME. (B) Senescence-inducing therapies give rise to a new cell subtype capable of modulating the phenotype of the other cells that make up the tumor niche (light blue cells); on the right, the most common types of cellular communication of SnCs are shown. (C) The heterogeneous composition of the tumor niche and, on the right, the main mechanisms modulated by the senescent cell. Abbreviations: SASP, senescence-associated secretory phenotype; SnCC, senescent cancer cell; TIS, therapy-induced senescence.

The induction of cellular senescence by numerous chemotherapeutics (CT) and radiotherapy began to be a target of attention from the observation that DNA damage generated by these therapies can lead some subclones of tumor cells to Therapy-Induced Senescence (TIS - i.e., senescence induced by radiotherapy or drugs like chemotherapeutics, microtubules inhibitors, targeted therapies, hormone receptor antagonists, among others) ( Acosta and Gil, 2012; Wang et al., 2022) ( Figure 1A and B). However, many studies evaluating TIS consider entering senescence as a terminal cell state, neglecting that, despite being non-proliferative, these cells remain metabolically active in the cellular composition of the tumor ( Collado and Serrano, 2010). In this way, through cellular mechanisms of communication like juxtacrine (i.e., a direct contact between cells) or paracrine (i.e., between nearby cells through soluble molecules) signaling, SnCs could interfere with the function of other non-SnCs in the TME, including non-senescent cancer cells (non-SnCCs), normal cells, immune cells, cancer stem cells (CSCs), endothelial cells, CAFs, and stromal cells ( Figure 1A and 1B) ( Yousefzadeh et al., 2021). Accordingly, SnCs could modulate several aspects related to tumor progression, such as cell proliferation, cell death, cell migration, angiogenesis, and resistance to therapy. Likewise, SnCs may also stimulate phenotypic transitions and unlock the cellular plasticity of other tumor and non-tumor cells from the TME ( Figure 1C) ( Wang *et al.,* 2022). 

In general, signaling mediated by SnCs in the TME involves both the secretion of soluble molecules and the direct contact with other cells through membrane nanotube formation or cell-cell contact. The myriad of components secreted to the extracellular environment by SnCs constitutes the so-called Senescence-Associated Secretory Phenotype (SASP), which is a chronic and dynamic feature that develops progressively over time from senescence triggering ( Coppé et al., 2008; Muñoz-Espín et al., 2013, Demaria et al., 2014; Alessio et al., 2023). SASP contains high levels of growth factors such as Epidermal Growth Factor (EGF) and Vascular Endothelial Growth Factor (VEGF) ( Strieter et al., 2006), transforming factors such as Gro-1 ( Yang et al., 2006), molecules that stimulate the EMT such as TGF-β ( Coppé *et al.,* 2008), enzymes that promote the invasion of adjacent tissues such as matrix metalloproteinases (MMPs) ( Liu and Hornsby, 2007; Ruhland et al., 2016), among others ( Figure 1B). In addition to these soluble molecules, SnCs can also secrete exosomes and microvesicles. These membrane-delimited extracellular vesicles can transport from small signaling molecules, proteins, and metabolites to messenger RNA, regulatory RNA, and even small pieces of DNA ( Wallis et al., 2020). In this way, SnCs (donors) can transfer molecules to other cells (acceptors)from neighbor cells in a paracrine manner to more distant cells through blood circulation ( Tkach and Thery, 2016). Through extracellular vesicles, therefore, SnCs can influence the behavior of other cells, modulate anticancer immunity, induce tumor progression, and facilitate the metastatic process ( Hoshino et al., 2015; Yokoi et al., 2017). 

Thus, TIS could initially reduce the speed of tumor growth by blocking cell proliferation in some susceptible neoplastic subclones. However, the long-term accumulation of SnCs could lead to an enrichment of SASP in the TME, favoring the proliferation, malignancy, and resistance of non-SnCCs ( Figure 1C). Indeed, recent studies suggest that patients with enrichment of senescent signatures have a worse prognosis for liver cancer ( Li and Xue, 2023; Zhang S et al., 2023), colorectal cancer ( Tan et al., 2023), glioma ( Yang et al., 2021), among others ( Zhang *et al.,* 2022) with rare exceptions showing the opposite ( Zhou et al., 2022).

Therefore, SnCs have two main phenotypic characteristics: 1) an autonomous feature characterized by loss of proliferative capacity and cellular fitness, culminating in the inability to leave descendants; and 2) a non-autonomous feature characterized by the secretion of soluble molecules and extracellular vesicles (SASP) and direct interaction with other cells. These two characteristics have different dominances considering carcinogenesis: the loss of proliferative capacity of some tumor cells, mainly due to senescence induced by oncogenes or loss of tumor suppressor genes, should contribute to preventing the proliferation of transformed cells and, therefore, the progression of early tumors. In these contexts, SnCCs should predominate to the detriment of proliferative cells. However, if in the microenvironment of early neoplasms, subclones with proliferative potential emerge, the second phenotypic characteristic of SnCs (i.e., their influence on other cells from the TME) may start to dominate and contribute to a gradual progression of the tumor, with a relative reduction in the number of SnCCs due to the proliferation of non-SnCCs. Thus, considering tumors that have already formed (and, therefore, have a greater chance of being diagnosed), proliferative cells are predominant about SnCs for evolutionary reasons. However, although proliferative cells are naturally resistant to senescence induced by changes in oncogenes and tumor suppressor genes, they can be sensitive to senescence induced by damage-inducing therapies. However, due to the well-established intratumoral heterogeneity, which can encompass dozens to hundreds of different tumor subclones, not all subclones are sensitive to TIS, as illustrated in Figure 1B. Thus, some subclones acquire a senescent phenotype and begin to secrete molecules promoting phenotypes that favor tumor progression, such as growth factors and extracellular matrix remodeling enzymes. Variables such as the number of subclones that will enter senescence, the constitution of the SASP of SnCCs, the phenotype of tumor cells that do not enter senescence, and the constitution of the TME may affect the prognosis of patients. However, to date, evidence suggests that regardless of these and other variables, the presence of SnCs in the TME of already-formed tumors appears to be associated with a worse prognosis, indicating a dominance of the pro-tumor (non-autonomous) effect of the senescent cell phenotype.

## Role of SnCs in tumor plasticity

### SnCs and epithelial to mesenchymal transition (EMT)

One of the most relevant mechanisms modulated by SASP is EMT, where tumor cells of epithelial origin acquire characteristics of mesenchymal cells, such as resistance to death induced by loss of cell adhesion, cell elongation, and greater migratory capacity ( Mittal, 2018). Therefore, despite the obligation of EMT in the process of metastasis has been questionable mainly because EMT is a spectrum of phenotypes rather than a binary event (Mittal, 2018; Lourenco et al., 2020), it may facilitate tumor malignancy and spread (Mittal, 2018). These events are clinically relevant since metastases are responsible for more than 80% of deaths associated with cancer ( Kalluri and Weinberg, 2009).

Through the secretion of Hepatocyte Growth Factor (HGF) and the activation of c-Met and MAPK in tumor cells, irradiation-induced fibroblasts promoted the migration and spread of pancreatic cancer cells ( Ohuchida et al., 2004). Likewise, in an animal model, the SASP from senescent CAFs promoted the peritoneal spread of gastric cancer by activating the JAK/STAT3 pathway in cancer cells ( Yasuda et al., 2021). Finally, senescent fibroblasts can also induce the migration of endothelial cells through the secretion of VEGF ( Coppé et al., 2006) and increase their contact with tumor cells, favoring angiogenesis and tumor development ( Orr and Wang, 2001). The promotion of EMT by conditioned medium containing SASP from senescent fibroblasts has also been observed in other tumor types such as breast ( Ortiz-Montero et al., 2017), prostate ( Bavik et al., 2006), bladder ( Goulet *et al.,* 2019) and ovary ( Lawrenson et al., 2010). 

As for the SASP produced by senescent fibroblasts, SASP derived from SnCCs is also able to promote EMT in non-SnCCs from breast and colorectal cancer ( Tato-Costa et al., 2016; Ortiz-Montero et al., 2017; Goulet *et al.,* 2019). Furthermore, SASP can also favor EMT and increased cell migration in pre-malignant cells ( Coppé et al., 2008; Lawrenson et al., 2010). This observation reinforces the hypothesis that the accumulation of SnCs in benign lesions could favor their malignancy, justifying the elimination of these cells as a mechanism to prevent tumor progression ( Choi et al., 2022; Kohli et al., 2022). Effector molecules from SASP involved in EMT promotion include interleukins like IL-6 ( Bavik et al., 2006) and IL-8 ( Bavik *et al.,* 2006; Tato-Costa *et al.,* 2016; Ortiz-Montero *et al.,* 2017), growth factors like HGF ( Ohuchida et al., 2004) and FGF-7 ( Bavik, Coleman *et al.,* 2006) and amphiregulin ( Bavik *et al.,* 2006). In this context, an essential translational aspect concerns the cell non-autonomous role of mutations in driver genes in the role of SASP. Mutations in the Ras oncogene or in TP53 enhance and accelerate the secretion of promalignant SASP in cells that have such alterations favoring EMT and TME remodeling, characterizing a non-autonomous effect of these mutations on tumor biology ( Coppé *et al.,* 2008).

Considering the intratumor heterogeneity, it is plausible to assume that some tumor subclones have differential sensitivity to TIS. In colorectal cancer cells subjected to 5-Fluorouracil (5-FU) treatment, specific subclones experienced apoptosis or underwent senescence, while others resisted to the drug. ( Cho et al., 2020; Baldasso-Zanon et al., 2024). In a condition like that, inferring that resistant clones are susceptible to the SASP from TIS cells is plausible. Indeed, in primary rectal cancer samples, the EMT markers are increased close to niches of SnCs compared to regions where SnCs are absent ( Tato-Costa et al., 2016). Corroborating that, ascites samples from metastatic gastric cancer patients presented an increase in fibroblasts producing proinflammatory SASP (i.e., IL-6, IL-8, VEGF, and others), compared to patients with no metastasis ( Yasuda et al., 2021). 

Translationally, another relevant aspect regarding the effect of SnCs on cancer concerns the clinical treatment protocol. For example, neoadjuvant therapies can lead to an enrichment of SnCs in the TME, as in the case of breast cancer treated with Doxorubicin ( Achuthan et al., 2011; Febres-Aldana et al., 2020; Saleh et al., 2021) or colorectal cancer treated with chemotherapy (5-FU or Doxorubicin) plus radiotherapy ( Tato-Costa et al., 2016). Consequently, although it initially reduces tumor growth rate, this enrichment could lead to a worse prognosis for patients in the long term. On the other hand, considering adjuvant therapy schedules, for most chemotherapy drugs, patients are exposed to multiple cycles of drug exposition, interspersed with periods of recovery for the patient. Thus, the first treatment cycle may lead to the enrichment of SnCs in the TME. In this context, patients exposed to senescence-inducing therapies could benefit from consecutive chemotherapy treatment followed by senolytic treatment to eliminate SnCs. This approach is promising since it is plausible to infer that SnCCs resist re-exposure to chemotherapy. However, there is no strong evidence on this issue. Finally, alternative chemotherapy protocols have emerged in recent years in which lower doses of chemotherapy drugs are used for more extended periods. However, this strategy may lead to an even more significant enrichment of the senescent population and inflammatory cytokine secretion in the TME ( Rodier et al., 2009) since intermediate DNA damage, for example, favors senescence over apoptosis, which requires higher rates of damage to be triggered ( Zhang et al., 2010).

In conclusion, multiple pieces of evidence converge to a clear role for SASP in promoting EMT and increasing aggressiveness in non-SnCCs. More than one cell type can undergo senescence in the TME after therapies. Therefore, the SASP origin can be tumor cells, fibroblasts, or other cell types. Consequently, despite the initial effect of therapies in controlling tumor growth through apoptosis and senescence, in the long term, SASP may induce EMT in those tolerant subclones, increasing tumor aggressiveness. This crosstalk may explain, at least partially, the association between high levels of senescence in the TME and worse clinical prognosis. Discovering the exact molecules responsible for the pro-tumor role played by SASP, as well as the tumor signaling pathways responsive to these molecules, is essential to allow the development of therapies that may inhibit both the production of these molecules by SnCs and the responsiveness of tumor cells to these signals.

### Cell differentiation and stemness

The balance between maintaining cells in an undifferentiated state and cell differentiation is disturbed in tumor biology. The clinical relevance of this aspect supports the classification of tumor grade, with poorly differentiated or undifferentiated tumors being classified as having a higher grade. The main cellular phenotype associated with stemness is cancer stem cells (CSCs), a subpopulation of cancer cells likely responsible for tumor initiation, growth, and recurrence. CSCs have intrinsic features like greater migratory capacity, more remarkable plasticity, greater capacity for invasiveness, and survival outside the primary focus ( Batlle and Clevers, 2017). In addition to these phenotypic hallmarks, CSCs express specific markers, allowing their identification in different tumor types. Considering the abovementioned characteristics, an enrichment of CSCs and stemness markers may suggest heightened aggressiveness and a less favorable prognosis ( Yang et al., 2020). 

The enrichment of SnCs in the TME led to increased CSCs and stemness markers, mainly through SASP molecules. In colorectal cancer, melanoma, and hematological malignancies, the enrichment of senescence markers was associated with increased classic stemness markers like CD133, CD44, LGR5, and CD34. The effect of SnCs in increasing population stemness may be mediated, at least partially, by the reprogramming of non-CSCs into CSC-like tumor cells, with increased tumor formation *in vivo* ( Milanovic et al., 2018). SnCs can also induce the dedifferentiation of tumor cells, thus providing more remarkable plasticity to transformed cells and resulting in the emergence and maintenance of a subpopulation of CSCs ( Cahu et al., 2012; Castro-Vega et al., 2015). Likewise, premalignant mammary epithelial cells exposed to senescent fibroblasts lose differentiated features and undergo malignant transformation. Furthermore, the injection of premalignant cells with senescent fibroblasts in mice accelerated tumor formation compared to injecting tumor cells alone ( Krtolica et al., 2001; Parrinello et al., 2005). Therefore, the accumulation of therapy-induced SnCs could promote, in the long term, the process of tumor resistance and recurrence, marked by the greater pharmacological tolerance of the CSCs, followed by the reacquisition of the proliferative capacity of the surviving subpopulation. Furthermore, in addition to TIS, OIS also seems to lead to a SASP that stimulates cell stemness and plasticity ( Ritschka et al., 2017).

In addition to the nonautonomous effects, cells with senescent features seem to release small aneuploid cells by unknown mechanisms. These small cells can re-enter the cell cycle and acquire a stem-like state through activating the Wnt pathway ( Milanovic et al., 2018). Although interesting, it is still not clear how this happens, whether the senescent cell can reprogram itself to acquire a stem state or whether the senescent cell can generate new cells through endoreplication or budding ( Zhang, et al., 2014; Czarnecka-Herok et al., 2022). This behavior is commonly observed in polyploid-giant cancer cells (PGCCs), which have features of SnCs and have been associated with tumor recurrence in several cancer types ( White-Gilbertson and Voelkel-Johnson, 2020). Complementary, using cellular models that allow SnCs to re-enter the cell cycle through genetic or epigenetic alterations, cells released from senescence showed more significant formation of colonies *in vitro* and tumors *in vivo* compared to those cells that had never undergone a senescent state ( Yang et al., 2017). However, it is important to mention that this is an artificial experimental model in which cells are forced to re-enter the cell cycle, with no concrete evidence showing the spontaneous reversibility of senescence in either physiological or pathological contexts.

**Figure 2 -:**
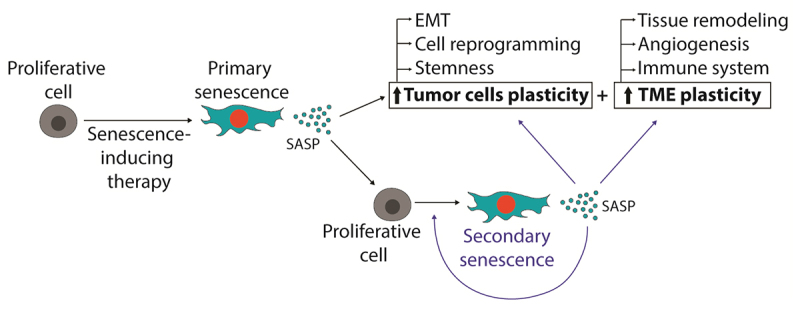
Positive feedback between senescence induction and increased tumor plasticity. After the induction of senescence by a chemotherapy drug (CT), there is an enrichment of SASP in the TME. Molecules in SASP such as IL-6, IL8, and TNF-α fuel the plasticity of tumor cells (including cell reprogramming, increased stemness, and epithelial-to-mesenchymal transition) and TME. Additionally, SASP molecules can induce more cells to enter a senescent state (secondary senescence), producing even more SASP. This creates a feedback loop that perpetuates increased plasticity and tumor heterogeneity. Abbreviations: EMT, epithelial-to-mesenchymal transition.

Molecularly, NF-kβ, a key factor controlling the transcription of proinflammatory molecules, had a central role in the effect of SnCs on increasing the population stemness. Likewise, pro-inflammatory molecules like IL-6 play a positive role in the maintenance of stemness capacity by CSCs ( Korkaya et al., 2011), which is closely related to the pro-stemness role induced by SnCs. Indeed, senescence-associated IL-6 and IL-8 increased the expression of CD44, a CSC marker, in breast cancer cells. Interestingly, the exposure to a senescence-conditioned medium also led tumor cells to the expression and secretion of IL-6 and IL-8 and the induction of senescence, thus building an autocrine pro-tumoral signaling ( Figure 2). Other soluble molecules from SASP, like TGF-β and VEGF, can also promote secondary senescence ( Matsuda et al., 2023). Promisingly, neutralizing these interleukins using specific antibodies can reverse the effects of a senescence-conditioned medium ( Ortiz-Montero et al., 2017).

Physiologically, maintaining cells in a stem state or cell reprogramming during tissue injury or development is essential for adaptive responses, tissue repair, and homeostasis. As raised, paracrine factors secreted by SnCs may be necessary to enrich these phenotypes. However, upon reacquisition of tissue homeostasis, this undifferentiated cellular phenotype needs to be reverted to a state of differentiation. In the same way that contexts of long-lasting tissue stress such as chronic inflammation predispose to cellular transformation, the presence of SnCs and its secreted molecules like proinflammatory interleukins for long periods could favor the maintenance of cells in an undifferentiated state that is more permissive to cellular transformation ( Yasuda et al., 2021), tumor progression or recurrence ( Demaria et al., 2017) ( Figure 2).

### SnCs and cell reprogramming

In addition to controlling cellular differentiation, genomic reprogramming is another central mechanism of cellular plasticity necessary for tissue homeostasis. Chronic disturbances in these two events are involved in tumor initiation, progression, and prognosis ( Ohnishi et al., 2014; Xiong et al., 2019). As raised in this section, initial evidence suggests that SnCs could modulate tumor cells’ reprogramming through autonomous and non-autonomous mechanisms. 

Most evidence of the role of SnCs in cellular reprogramming comes from tissue damage and repair models. The tissue damage and repair microenvironment share many characteristics observed in the TME, including inflammatory cells and molecules, extracellular matrix remodeling, intense paracrine signaling, angiogenesis, cell death, and proliferation ( Jin and Jin, 2020). Stress-induced SnCs favor *in vivo* reparative cellular reprogramming in skeletal muscle fibers ( Chiche et al., 2017) and gastric, pancreatic, and kidney epithelial cells ( Mosteiro et al., 2016). This effect is exerted mainly through molecules present in the SASP, such as IL-6 ( Mosteiro *et al.,* 2016; Chiche *et al.,* 2017; Mosteiro *et al.,* 2018) and TNF-α ( Mosteiro *et al.,* 2016), whose production is mediated by the INK4/Arf locus. These factors can modulate the expression and activity of effector molecules, mainly transcription factors such as Nanog ( Mosteiro *et al.,* 2016; Chiche *et al.,* 2017). However, the effect of SASP on cell differentiation or reprogramming seems to depend, among other variables, on the time of exposure to SASP. In keratinocytes, for example, transient exposure to SASP promoted an increase in the expression of stem cell markers. On the other hand, prolonged exposure to SASP induced senescence in these cells, with a loss of regenerative capacity ( Ritschka et al., 2017). Furthermore, the reduction of SnCs by genetic clearance (i.e., the selective killing of cells with specific genetic features using a gene construct, ( Baker et al., 2011) of cells expressing high levels of p16 or by using senotherapeutics (i.e., a class of drugs or interventions aimed at targeting and eliminating SnCs, ( Zhang L et al., 2023) increased *in vitro* and *in vivo* somatic reprogramming. These strategies also increased the establishment of induced Pluripotent Stem Cells (iPSC), a type of stem cell that is artificially generated from non-pluripotent somatic cells ( Takahashi and Yamanaka, 2006), as well as histopathological features of tissue regeneration in the liver ( Grigorash et al., 2023). Corroborating this, chronic SASP led to reduced stemness in intestine organoids and impaired crypt formation, an effect that was mediated by secreted PTK7 present in the SASP composition ( Yun et al., 2023).

As raised, the transient enrichment of SnCs in human tissues in response to local signals is beneficial and contributes to maintaining or reestablishing tissue homeostasis. On the other hand, its enrichment induced by therapies (i.e., from exogenous stress to the body) in the TME is usually associated with a worse prognosis and tumor progression ( Domen et al., 2022; Zhang S et al., 2023). This deleterious effect of SnCs on the TME may be related to a) the presence of SnCs for more extended periods than those observed in contexts of tissue development and repair; b) the accumulation of SnCs, exceeding the beneficial threshold and leading to deleterious effects; c) the composition of SASP produced by SnCCs cells, which may differ from the SASP produced in other pathophysiological responses. In the TME, the presence of SnCs, even for a short time, could fuel the plasticity of some tumor subclones through the modulation of cellular reprogramming, differentiation, EMT, among others. Therefore, this may lead to increased tumor heterogeneity or capacity to adapt to stresses such as anti-tumor therapies ( Castro-Vega et al., 2015). This crosstalk reinforces the senoprevention arm (i.e., preventing cells from entering senescence, directing them to cell death) as the best therapeutic strategy related to the modulation of cellular senescence.

As raised, data from other human physiopathological responses has provided evidence that allows us to extrapolate the results to tumor biology, supporting translationally relevant hypotheses. Multiple factors secreted by SnCs can modulate the phenotypic plasticity of tumor cells. Among the biggest challenges in the area, it is necessary to understand what these factors are, how to modulate its production by SnCs, and how to act specifically on SnCs present in the TME without affecting SnCs involved in physiological responses critical for homeostasis such as tissue repair and remodeling.

## Survival mechanisms of senescent tumor cells

The state of cell proliferation arrest observed in SnCs arises from the integration of molecular signals that not only block cell cycle progression but also ensure that these cells remain alive. This phenotype is mediated by a complex network of pro-survival molecular signals known as Senescent-Cell Anti-apoptotic Pathways (SCAPs) ( Zhu et al., 2015). Among these pathways, the Bcl-2 family, MDM-2/TP53/p21, and PI3K/AKT/mTOR pathways are the most extensively investigated for their role in governing the survival of SnCCs ( Table 1).

**Table 1 -:** Survival pathways of SnCs involving Bcl-2 family protein, PI3K/AKT, and the MDM-2/TP53 pathways.

Author	Year	Molecular Marker	Molecular Status	Pre or Post Senescence	Main Outcome	Model
Bcl-2 family pathway						
Drullion C	2012	Bcl-2	Overexpression	Pre	Bcl-2 upregulation blocked apoptosis and increased levels of senescence in response to Imatinib.	Leukemia
Ikezawa K	2017	Bcl-X(L)	Downregulation	Pre	Downregulation of Bcl-X(L) by siRNA triggered oncogene-induced senescence in high-grade tumors, associated with p21 overexpression.	Pancreas tumor
Gayle S	2019	Bcl-X(L)		Pre	Bcl-X(L) was overexpressed in those cell lines that triggered senescence in response to BETi treatment. Bcl-2, Bim, BAX, and Mcl-1 levels were not changed in senescent cells.	Breast Cancer
Gayle S	2019	Bcl-X(L)	Overexpression	Pre	Bcl-X(L) overexpression in BETi-treated cells shifted the response from apoptosis to senescence.	Breast Cancer
Gayle S	2019	Bcl-X(L)	Downregulation	Pre/post	Bcl-X(L) inhibition induced apoptosis in response to BETi even after BETi-induced senescence had already occurred.	Breast Cancer
Selt F	2023	Bcl-X(L)		Pre	Oncogene-induced senescence increased Bcl-X(L) expression. Modulation of other anti-apoptotic Bcl-2 family proteins were not detected.	Pilocytic Astrocitoma
Selt F	2023	Bcl-X(L)	Downregulation	Post	Downregulation of Bcl-X(L) (Navitoclax or A-1331852) reduced the viability of senescent cells by apoptosis triggering. Bcl-2 and Mcl-1 inhibitors (Venetoclax and S63845) did not impact the viability of senescent cells.	Pilocytic Astrocitoma
Choi J	2018	Bcl-W	Overexpressison	Pre	Overexpression of Bcl-W promoted premature senescence by activating the TP53 pathway, increasing TP53, p21, Notch2 and p16^INK4A^.	Glioblastoma and Lung Cancer
Choi J	2018	Bcl-W	Downregulation	Pre	Downregulation of Bcl-W using miR-95-5p decreased premature senescence by suppressing Bcl-W and p21 expression.	Glioblastoma and Lung Cancer
Bolesta E	2012	Mcl-1	Overexpression	Pre	Overexpression of Mcl-1 before treatment abrogates the doxorubicin-induced senescence in TP53+ cells, reducing p21	Human cancers
Bolesta E	2012	Mcl-1	Downregulation	Pre	Downregulation of Mcl-1 before treatment triggered doxorubicin-induced senescence in TP53- cells, increasing p21	Colon cancer
Wu G	2022	Bid and BAX	Release from inhibitor	Post	The BH3 mimetic A-1331852 induced caspase-dependent senescent cell death by releasing Bid and BAX through disrupting Bcl-X(L)/Bid and Bcl-X(L)/BAX complexes.	Human Lung carcinoma
Werner L	2015	BAX		Post	Senescence induced by irradiation plus MDM-2 inhibitor induced TP53 accumulation, followed by increase in p21 and BAX.	Melanoma and Sarcoma
Drullion C	2012	Bim	Downregulation	Post	Blocking apoptosis by Bim downregulation increased senescence levels.	Leukemia
PI3K/AKT pathway						
Xu X	2014	AKT	Allosteric inhibition	Pre	Administration of AKT inhibitor (MK-2206) induces senescence through increasing ROS production and miR-182 expression, accompanied by an increase in TP53, p21 and p16^INK4A^	Leiomyoma
Jung S	2019	PTEN	Downregulation	Pre	PTEN downregulation by RNAi induces senescence through ATK-mTORC1/2 activation, followed by activation of the TP53-p21 axis, independently of DDR and ROS generation.	Breast cancer
MDM-2/TP53 pathway						
Yosef R	2017	p21	Downregulation	Post	Knockdown of p21 by RNAi in DNA damage-induced senescent cells induced DNA lesions, resulting in cell death through ATM and NF-kβ activation.	Non-Small Cell Lung Carcinoma

### Bcl-2 family pathway

Resistance to programmed cell death is a recognized hallmark of cancer, characterized by an altered molecular profile featuring heightened expression or activity of anti-apoptotic proteins, often accompanied by a reduction in the function of pro-apoptotic proteins ( Hanahan, 2022). This altered pattern of apoptotic proteins directly contributes to the survival of tumor cells and plays a pivotal role in triggering and maintaining the senescent state in cancer. One of the leading protein families involved in resistance to programmed cell death is the Bcl-2 family, which is constituted by pro-apoptotic and anti-apoptotic members responsible mainly for modulating the triggering of the intrinsic apoptosis pathway. However, each Bcl-2 family member has also distinct influences on non-canonical molecular mechanisms such as cellular senescence ( Fan et al., 2020). The expression level of individual Bcl-2 family proteins can affect the initiation of senescence differently. Once cells become senescent, members of the Bcl-2 family can be differently modulated in SnCs, not only enhancing the resistance to programmed cell death but also sustaining their permanent cell cycle arrest state ( Basu, 2022).

Bcl-2 family anti-apoptotic proteins

Anti-apoptotic proteins from the Bcl-2 family are often overexpressed in several tumor types, favoring the resistance to programmed cell death ( Kaloni et al., 2023). However, despite belonging to the same subgroup, each anti-apoptotic protein from the Bcl-2 family seems to influence both the triggering and the maintenance of the senescent state in tumor cells in a particular way, as depicted below and shown in Table 1.

Bcl-2 and Bcl-X(L): these Bcl-2 anti-apoptotic proteins play a crucial role in governing the delicate balance between cell life and death by avoiding the release of apoptotic factors throughout the mitochondrial outer membrane. In addition to their negative role in programmed cell death, both proteins control the initiation of cell cycle arrest, thus blocking apoptosis and simultaneously triggering TIS or OIS ( Drullion et al., 2012; Gayle et al., 2019). 

Moreover, the overexpression of Bcl-X(L) in tumor cells before treatment can attenuate apoptosis induction, favoring senescence triggering ( Gayle et al., 2019). These pieces of evidence show how important Bcl-2 and Bcl-X(L) are for the senescence initiation process, and their expression levels are definitive in determining the fate of a tumor cell in response to some types of therapy. For this reason, pharmacological inhibitors targeting these proteins, such as ABT263 (Navitoclax), have been using to eliminate SnCs, as discussed in the next section. 

On the other hand, despite demonstrating a similar effect on initiating senescence, Bcl-2 and Bcl-X(L) seem to have different relevance in its maintenance. Pharmacological or molecular inhibition of Bcl-X(L) can induce SnCCs to apoptosis (Selt. However, Venetoclax, a specific inhibitor of Bcl-2, did not impact the viability of established SnCs (Selt *et al.,* 2023), suggesting that Bcl-X(L) may be more critical not only for inducing but also for maintaining the senescent state. Controversially, some evidence show that downregulating Bcl-X(L) can also trigger senescence, but accompanied by an increase in the expression of the cell cycle inhibitor p21 ( Ikezawa et al., 2017). Therefore, the triggering of the senescent state may be a consequence of p21accumulation rather than the reduction of Bcl-X(L). 

Mcl-1: another anti-apoptotic member of the Bcl-2 family, Mcl-1 is also responsible for controlling the mitochondrial permeability, preventing the release of pro-apoptotic molecules from the mitochondrial inner space. However, the interplay between Mcl-1 and senescence in tumor biology is complex and controversial. While Mcl-1 can contribute to the survival of SnCCs and non-SnCCs by preventing apoptosis, its overexpression, unlike the above-discussed anti-apoptotic proteins, may also be associated with the evasion of senescence, allowing cancer cells to persist with their proliferation potential ( Bolesta et al., 2012). In the same way, the downregulation of Mcl-1 may allow the triggering of senescence in cancer cells ( Bolesta *et al.,* 2012). However, once senescent, the inhibition of this protein does not affect the viability of SnCCs ( Selt et al., 2023), showing its importance for initiating the process but not for its maintenance. 

Bcl-W: little is known about the involvement of Bcl-W in the induction or maintenance of the senescent state in tumor cells. Similarly to Bcl-X(L), one of the few pieces of evidence showed that Bcl-W overexpression also increases the induction of senescence in cancer cells, and its suppression by miRNA attenuates the permanent arrest in the cell cycle ( Choi et al., 2018). Although it is also a protein responsible for negatively controlling the permeability of the mitochondrial outer membrane, thus preventing the triggering of apoptosis, more evidence is needed to define its actual involvement in the triggering and maintenance of senescence.

Although there are other anti-apoptotic proteins in the composition of the Bcl-2 family, there is no evidence about their direct involvement in regulating senescence in cancer. Therefore, it is possible to define that the involvement of Bcl-2 family anti-apoptotic proteins seems to depend not only on their anti-apoptotic properties but also on its influence in modulating the expression and activity of other proteins responsible to modulate the cell cycle arrest, such as p21 protein. Classifying proteins from the Bcl-2 family as anti-apoptotic molecules does not necessarily imply assigning them a role as senescence-inducing proteins, which must be considered in developing senolytic therapies.

Bcl-2 family pro-apoptotic proteins

Pro-apoptotic proteins within the Bcl-2 family also modulate the initiation and maintenance of senescence in tumor cells. These proteins are usually found at reduced levels or in stoichiometric imbalance with anti-apoptotic proteins in tumor cells, hampering apoptosis and potentially influencing senescence. However, there is little evidence correlating and explaining how the expression levels or activity of pro-apoptotic proteins of the Bcl-2 family can modulate the senescent state in cancer.

Bim: the Bcl-2 interacting mediator of cell death (Bim) is a pro-apoptotic protein that initiates apoptosis by binding to and neutralizing anti-apoptotic Bcl-2 proteins, such as Bcl-2, Bcl-X(L), and Mcl-1. Through these interactions, BIM contributes to releasing pro-apoptotic factors from the inner space of mitochondria, leading to the activation of caspases, and ultimately to cell death ( Kale et al., 2018). Likewise, reducing the expression level of Bim inhibits apoptosis while increases senescence in human leukemia cells ( Drullion et al., 2012). Nonetheless, little evidence explains how Bim can favor senescence.

Bid and BAX: Bid serves as a BH3-only protein and links the intrinsic and extrinsic apoptotic pathways. Upon activation, Bid triggers mitochondrial outer membrane permeabilization, contributing to the release of pro-apoptotic factors. On the other hand, BAX is a multi-domain pro-apoptotic protein that plays a crucial role in regulating mitochondrial integrity. Activated BAX undergoes conformational changes and translocates to the mitochondrial outer membrane, where it promotes permeabilization through the formation of pores, allowing the release of apoptogenic factors from the inner space of this organelle, ultimately leading to cell death. Thus, cell survival depends on a stoichiometric balance between anti- and pro-apoptotic proteins of this family, regulating the apoptosis process through an inhibitory physical interaction of anti-apoptotic protein units with pro-apoptotic proteins. BH3 mimetic molecules like A-1331852, which function as a subgroup of proteins known as pro-apoptotic BH3-only proteins, induce apoptosis in senescent lung carcinoma cells by disrupting the Bcl-X(L)-mediated inhibitory interaction with pro-apoptotic proteins Bid and BAX, ( Wu et al., 2022). However, increasing pro-apoptotic proteins like BAX does not prevent the senescent state in tumor cells, whether, at the same time, senescence inducer proteins such as cell cycle inhibitor p21 are being simultaneously increased ( Werner et al., 2015), suggesting that apoptosis inducers are not enough to block the triggering of the senescent state.

### MDM-2/TP53/p21 pathway

Another extensively studied SCAP in cancer is the MDM-2/TP53/p21 signaling pathway, which is responsible for sensing DNA damage caused by several stress signals and inducing cell cycle arrest for DNA repair. Persistent DNA damage signals activate TP53, a transcription factor for several target genes that control cell cycle arrest, apoptosis, and senescence. Despite the wide variety of molecular targets modulated by TP53, the induction of CDKN1A gene, which encodes the p21 protein, represents the main contribution of TP53 in triggering senescence and preserving cell survival. As a member of the CDK inhibitor family, p21 mediates the expression of several molecular targets that result in cell cycle arrest at either the G1/S or G2/M checkpoints ( Rufini et al., 2013; Al Bitar and Gali-Muhtasib, 2019). However, p21 seems to have an even greater relevance as an effector molecule in the dual decision between apoptosis or senescence in response to stress. In addition to inducing cell cycle arrest, p21 can also interact with Bcl-2 proteins and inhibit apoptosis, reinforcing the decision for senescence ( Martinez et al., 2002; Yosef et al., 2017). In SnCCs resulting from DNA damage, the downregulation of p21 can trigger cell death through ATM and NF-kβ activation, highlighting the significance of high levels of p21 to maintaining the senescent state ( Yosef *et al.,* 2017). 

### p16 pathway

In addition to the action of p21, which seems to be important in the senescence initiation process ( Kuilman et al., 2010), another crucial factor for this cellular outcome in cancer is p16, which seems to be more involved in the senescence maintenance in a protein level-dependent manner ( Rayess et al., 2012). Currently, p16 is considered a tumor suppressor protein because of its physiological role as a cell cycle inhibitor and its downregulated expression in many tumors. Intriguingly, overexpression of p16 has also been described in several tumors ( Romagosa et al., 2011). The central role of p16 is to inhibit the cyclin D1/CDK4/6 complex, preventing the hyperphosphorylation of the Rb protein. This event allows the release of E2F to mediate the expression of effector molecules controlling the progression of the cell cycle phase G1 for S phase ( Peurala et al., 2013). Thus, high levels of p16 seem essential in maintaining the permanent cell cycle arrest present in SnCs. The suppression of p16 in SnCs could reverse the cell cycle arrest only when p53 was also inactivated since p21 can compensate for maintaining the senescent state. Likewise, once p16 is highly expressed, the downregulation of TP53 cannot reverse the cell cycle arrest ( Campisi, 2005), suggesting that p16 seems to be as relevant as p21 for the triggering and maintenance of the senescent state. Therefore, although p21 can directly participate in cell survival through the inhibitory modulation of apoptosis, together with p16 it is fundamental for maintaining the senescent state.

### PI3K/AKT/mTOR pathway

The PI3K/Akt/mTOR pathway, an intricate signaling cascade, is pivotal in regulating various cellular processes, including cell growth, survival, and senescence. This pathway is tightly regulated and plays a crucial role in normal cellular functions, and its dysregulation is commonly observed in various diseases, particularly cancer. Hyperactivation of this pathway is associated with uncontrolled cell growth, evasion of apoptosis, and resistance to anti-cancer therapies. However, although much is known about the dysregulation of the PI3K/Akt/mTOR pathway in the context of cancer, there is little evidence directly correlating its modulation with survival, specifically in SnCCs. It is known that it can also regulate p21 expression to initiate senescence in tumor cells, either in a DNA damage response-dependent ( Xu et al., 2014) or independent manner ( Jung et al., 2019). Thus, more evidence is needed to define the actual involvement of this signaling pathway in modulating the survival of SnCs.

## Senotherapies in cancer: From senoprevention to senolysis

**Figure 3 -:**
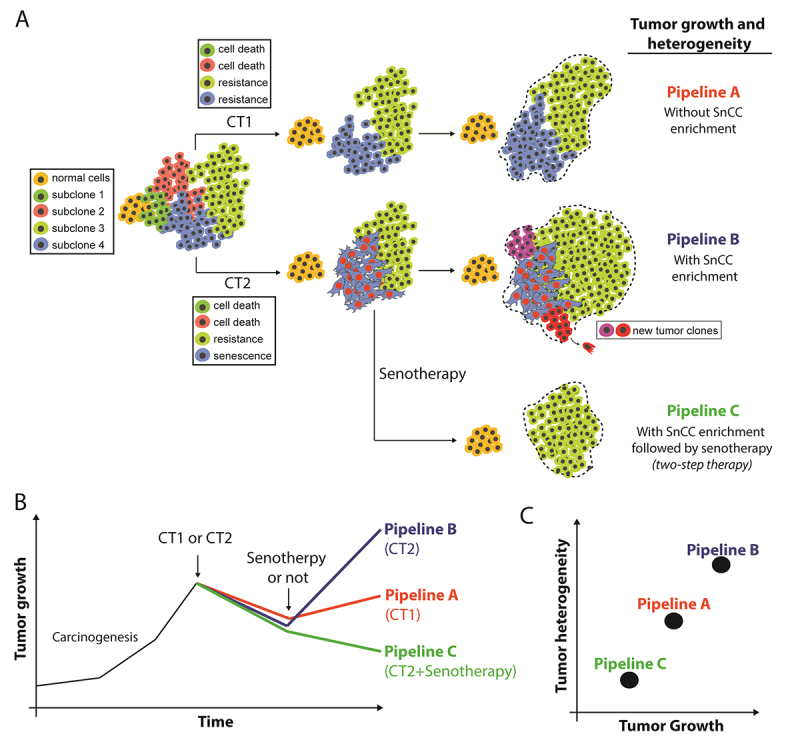
The impact of Senescent Cells (SnCs) and senotherapies in tumor growth. A). Representative model of the parenchyma of a tumor formed by 4 subclones of tumor cells and 1 subclone of a normal cell. **Top (pipeline A)** - cell response to chemotherapy 1 (CT1) considering 2 tumor clones sensitive to cell death and 2 tumor clones resistant to therapy. Note that there is no induction of senescence in this example. **Bottom (pipeline B)** - response to chemotherapy 2 (CT2) considering 2 tumor clones sensitive to cell death, 1 tumor clone resistant to therapy and 1 clone sensitive to entry into a senescent state. Note that there is an enrichment of SnCs in this example, with increased heterogeneity and greater tumor growth compared to the Pipeline A model. **Pipeline C** represents two-step therapy, in which after the enrichment of SnCs in the TME, there is treatment with a senolytic compound that induces SnCs to cell death. Note that this is the treatment pipeline with the smallest tumor size at the end of the model. **B)** Growth curve simulations for pipelines A, B, and C. **C)** Scatter plot of heterogeneity and tumor growth for pipelines A, B, and C. Abbreviations: CT, chemotherapeutics; SnCC, senescent cancer cell.

Since the persistence of SnCs in the TME may favor tumor growth, increased heterogeneity, and resistance to therapy ( Krtolica et al., 2001; Castro-Vega et al., 2015), removing SnCs could reduce these pro-tumoral phenotypes ( Figure 3B and 3C), improving the prognosis ( Sieben et al., 2018). In recent years, researchers have developed a range of molecules to target SnCs, known as senotherapies. Many of these molecular signaling pathways targeted by senotherapeutics are crucial for the survival of SnCs, resulting in the senolysis. Preliminary evidence indicates that these compounds can eliminate SnCs *in vivo* ( Ellison-Hughes, 2020), leading to decreased inflammation, improved organ and tissue function, and ultimately increased survival in animal models ( Xu et al., 2018; Lewis-McDougall et al., 2019; Novais et al., 2021).

As raised in previous section, numerous mechanisms contributing to the resistance to apoptosis and the survival of SnCs have been identified ( Table 1). Based on these findings, researchers have investigated pharmacological inhibitors of these pathways for their potential to induce senescent cell death. Indeed, compounds that target anti-apoptotic proteins from the Bcl-2 family exhibit senolytic properties. For instance, ABT-263 (Navitoclax), a specific Bcl-2 inhibitor, and ABT-737, an inhibitor of Bcl-W, Bcl-X(L), and Mcl-1, have shown senolytic effects ( Yosef et al., 2016; Zhu et al., 2016). Fisetin, a specific Bcl-X(L) inhibitor, has demonstrated similar properties ( Yousefzadeh et al., 2018). Quercetin also triggers senescent cell death by interfering with the Bcl-X(L) protein, with more significant results observed when combined with Dasatinib ( Islam et al., 2023). Additionally, mTOR inhibitors, such as AZD8055, have shown senolytic effects inducing apoptosis by modulation of Bcl-2 family proteins ( Sharma et al., 2014). Cardiac glycosides have also displayed senolytic potential by affecting the pro-apoptotic Bcl-2 family protein NOX activator 1 ( Guerrero et al., 2019). 

Other senolytics that operate putatively independently of PI3K and mTOR pathways have been explored. For example, ARV825, a hetero-bifunctional proteolysis-targeting chimera, reduces *XRCC4* gene expression, hinders the recruitment of p53-binding protein 1 (53BP1), disrupts non-homologous end-joining DNA repair, and triggers apoptosis ( Wang et al., 2022). As an alternative approach, researchers have explored cellular senotherapies using Chimeric Antigen Receptor T-cells (CAR-T) targeting surface proteins expressed by SnCs, such as the PLAUR protein ( Amor et al., 2020).

Furthermore, oxidative phosphorylation is another molecular process that significantly influences the survival of SnCs. Its inhibitors (e.g. metformin) could sensitize SnCs while also acting as senomorphics, another class of senotherapeutics capable of altering the composition of the SASP through the modulation of intracellular pathways ( Cheng et al., 2022). Molecules with senomorphic properties have become increasingly relevant since signaling mediated by some proteins encoded by oncogenes can also modulate the production of SASP. Some examples are MEK1/2 ( Ruscetti et al., 2018, 2020), tyrosine kinase receptors such as EGFR ( Alexander et al., 2015; Romaniello et al., 2022), and the mTOR kinase ( Garbers et al., 2013; Herranz et al., 2015; Laberge et al., 2015) for which targeted therapies are approved. Indeed, the EGFR inhibitor erlotinib ( Alexander *et al.,* 2015), the MEK1/2 inhibitor trametinib ( Schick et al., 2015), and mTOR inhibitors (Herranz *et al.,* 2015) change the production and secretion of SASP molecules by SnCs. Thus, the genetic status of a given cancer and the targeted drugs chosen to treat it may affect the constitution of SASP, characterizing them as senomorphic molecules. However, additional evidence is needed to support this evidence in vivo. Finally, although these pathways can affect cell survival in human cells in general, whether the status of these oncogenes affects the sensitivity to analytics is still being determined.

Notably, SnCs may exhibit a reduced response to typical chemotherapy, which targets DNA replication in the S phase. They also frequently present heightened anti-apoptotic proteins and drug resistance, aiding their survival during subsequent cycles of treatments, which is the standard practice in the clinics. Thus, the rational combination of antineoplastic and senolytic therapies can mitigate SnCs’ pro-tumor effects. Considering that senescence also plays physiological roles like development and tissue repair, it is essential to understand the peculiarities of each type of senescence induced by different types of stimuli from different origins or contexts to enable the selective elimination of SnCCs from TME without affecting the beneficial roles that the physiological senescence perform in other biological situations.

From an evolutionary perspective, senescence can act as a tumor suppressor mechanism, ensuring successful reproduction in young individuals despite potential drawbacks later in life. However, it may become detrimental in older organisms. This is evident from the accumulation of SnCs in older organisms and their presence at sites of age-related pathologies, including cancer. However, there is a lack of quantitative data stratifying SnCs in age-related cancers before and after therapy-inducing senescence. Notwithstanding, there is a recognized understanding of age-related differences in tumors. For example, in breast cancer, older patients exhibit variations in tumor histology based on age ( Schonberg et al., 2010; Lodi et al., 2017; Wang et al., 2018), distinct subtype distributions ( Dreyer et al., 2013; Jenkins et al., 2014), and age-specific patterns of tumor mutations in comparison to younger patients ( Syed et al., 2014). Likewise, some studies suggest significant changes in tumor-infiltrating immune cells between tumors in older versus younger individuals ( Thomas et al., 2013; Chen et al., 2015). However, whether increased SnCs in older people mediate these differences is unknown. Therefore, the distinct TME between young and old patients suggests that anti-cancer therapy inducing senescence may not yield similar outcomes across age groups. Furthermore, it is plausible to infer that cells from older individuals would be more ‘primed’ for senescence (e.g., with increased basal histone H2AX signal) so that senescence could be induced by lower levels of damage than necessary for induce senescence in cells of younger people. However, it is essential to point out that most human cancers present cells with overexpression of the telomerase, which allows them to evade replicative senescence and attenuate the signals that would make them ‘primed’ for senescence.

Finally, another clinical characteristic associated with aging that may impact the role of senescence in some tumor types concerns the deregulation of hormone production or signaling ( Khosla et al., 2020). In certain hormone-related cancers, like from breast and prostate, a crosstalk between therapies targeting hormone receptors or signaling pathways and senescence has been proposed. By using a combination of drugs inhibiting Human Epidermal growth factor Receptor 2 (HER2) and Rb checkpoint, Viganò et al. (2022) showed that breast cancer cells exposed to these drugs undergo senescence ( Viganò *et al.,* 2022). Other studies corroborate the observation of senescence induction in breast and prostate cancer cells exposed to hormone-receptor antagonists tamoxifen ( Lee et al., 2014) and bicalutamide ( Carpenter et al., 2021), or androgen deprivation ( Ewald et al., 2013). Likewise, data from patients with ER+ and HER2+ breast cancer enrolled in a clinical trial showed that exposure to a combination of drugs that target these molecules presented a higher expression of senescence-related genes ( Viganò *et al.,* 2022). These data reinforce the complexity of senescence in pathophysiological contexts such as endocrine regulation, which is affected not only in numerous types of cancer but also during aging and other metabolic diseases. Therefore, the dysregulation of the crosstalk between hormonal regulation, aging, and cellular transformation may not only be the target of therapies but also underlie the pathogenesis of tumors of an endocrine nature associated with aging.

## Open questions and perspectives

**Figure 4 -:**
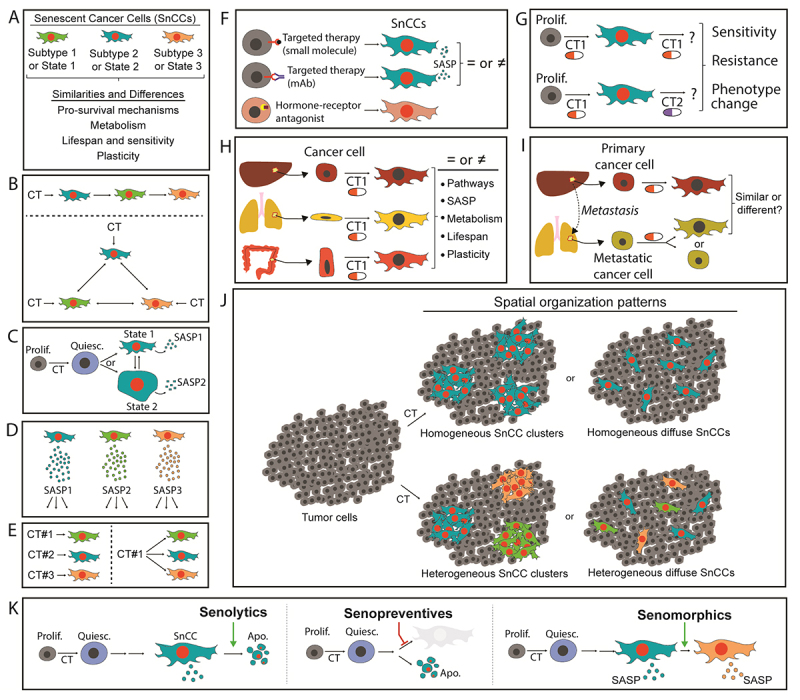
Open questions and perspectives. This figure summarizes questions that remain open about the biology of senescent tumor cells, especially related to therapy-induced senescence. **A)** It is not known how many senescent subtypes (or states) are induced after therapy, and differences or similarities between them. **B)** Considering initial evidence that points to phenotypic heterogeneity among tumor cells, it is not clear whether the same senescent cell is plastic enough to assume different subtypes (or states), nor whether a chemotherapeutic drug can induce different subtypes or states of senescence (e.g., from tumor cells with different molecular backgrounds). **C)** Although it is possible to observe morphological heterogeneity between SnCs, it is not clear whether the same senescent cell can transition between different subtypes or phenotypic states. **D)** SnCs of different subtypes (or states) may have qualitative and quantitative differences in SASP. **E)** It is not clear whether different chemotherapeutic agents induce SnCs of different subtypes or states, nor whether the same chemotherapy agent can induce SnCs with different subtypes or phenotypic states. **F)** Other classes of anti-cancer drugs, such as targeted therapies and hormone receptor antagonists, can also induce senescence. **G)** The sensitivity of cells induced to senescence by different chemotherapeutic agents is not known, both for the same chemotherapy agent that primarily induced senescence (top) and for other chemotherapy agents (cross-sensitivity, bottom). **H)** It is well established that tumor cells from different organs undergo cellular senescence after therapy. However, similarities and differences in the phenotype of these cells are not known. **I)** Comparison between SnCs derived from related primary and metastatic tumors. Differences and similarities between these cells are not known. **J)** Possible patterns of spatial organization of SnCs in the TME. SnCs, whether homogeneous among themselves or not, appear in clusters or diffuse. **K)** Main strategies targeting SnCs. Top - senolysis (induction of death of SnCs); middle - senoprevention (prevent cells from entering senescence, inducing them to cell death); bottom - senomorphics (modulation of SASP composition). Abbreviations: Apo., apoptotic cell; CT, chemotherapeutics; mAB, monoclonal antibody; Prolif., proliferative cell; Quiesc., quiescent cell.

Despite the advances made in recent years, many questions related to SnCCs and their impact on the TME remain open. Considering the biology of these transformed cells, it is necessary to characterize:

1. Differences and similarities between SnCs and SnCCs as well as the heterogeneity of SnCCs, considering SnCCs subtypes (or subpopulations) or states ( Figure 4A).
2. Whether the progressive acquisition of the senescent phenotype is unidirectional and irreversible ( Figure 4B - top) or if SnCCs can assume different states in a dynamic manner ( Figure 4B - bottom).
3. The morphological plasticity of SnCCs ( Figure 4C), including mechanisms controlling this process.
4. The SASP of different subtypes or states of SnCCs ( Figure 4D), which may influence the impact of these cells in the TME.

In addition to the biology of SnCs, some aspects related to senescence induced by antitumor therapies also need to be better understood, such as:

1. Differences between SnCCs induced by distinct chemotherapeutics (CT) ( Figure 4E - left) and whether a pro-senescent CT induces multiple or a specific SnCC subtype ( Figure 4E - right). Also, the characteristics of SnCCs induced by targeted or endocrine therapies ( Figure 4F).
2. How SnCCs respond to the re-treatment with the CT that induced the phenotype or to another CT ( Figure 4G).
3. Differences and similarities between SnCCs from different tumor types ( Figure 4H) or between SnCCs from primary and metastatic tumors ( Figure 4I).
4. The spatial organization of SnCCs in the TME (e.g. if these cells form niches or if they present a diffuse pattern of distribution) ( Figure 4F).
5. What is the best strategy of senotherapy to attenuate the pro-tumor effects played by SnCCs in the TME ( Figure 4H).

Although molecular biology tools have advanced enormously, most of the data regarding the role of SnCC in tumor plasticity still come from *in vitro* studies using limited and simplistic models based on cell population data. In contrast, data from primary samples are still limited. Furthermore, much evidence is based on tissue bulk data, making it difficult to determine which cell type in the sample undergoes senescence (e.g., tumor cells or stromal cells) and contributes to the biological effects of SnCs. Finally, much evidence comes from end-point analysis, which hampers conclusions about phenotypic plasticity and dynamics ( Begnini et al., 2022). Finding answers to the above questions will require strategies combining cellular and molecular tools, in addition to the development of new models and protocols, especially for live single-cell tracking and *in vivo* models. 

The presence of SnCs in the TME and unlocking phenotypic plasticity are the two most recent features included as hallmarks of cancer ( Hanahan, 2022). As discussed throughout this article, the first seems to influence the second strongly. The mechanisms of cellular plasticity modulated by SnCs are interconnected and are, to a certain extent, interdependent. Changes in nuclear gene expression, for example, are fundamental not only for stem cell differentiation and cellular reprogramming but also for phenotypic changes observed in EMT or metabolic changes. Furthermore, in some models, especially considering highly heterogeneous tumor populations, SnCCs may affect different cellular plasticity mechanisms depending on the background of the target cell. This explains, at least in part, the multiple phenotypic responses, like increased secondary senescence, stemness, and EMT, observed in the same tumor cell population *in vitro* after exposure to a SnCs-conditioned medium. Furthermore, more than one phenotype associated with cellular plasticity, such as increased stemness, EMT, and greater migratory capacity, can occur in the same cell after exposure to SASP ( Parrinello et al., 2005). Finally, it is essential to highlight that SASP can induce more cells to senescence (secondary senescence), promoting the persistence of SASP effects ( Figure 2).

As raised throughout the article, SnCs may contribute to TME remodeling during tumor formation and progression through multiple cell communication mechanisms, affecting the spatial organization and the functional status of stromal and immune cells. Most of the effects played by SnCs on mechanisms associated with tumor plasticity are exerted by the SASP. Thus, both the neutralization of SASP molecules and the modulation of intracellular pathways involved in SASP production are potential targets for therapies ( Ortiz-Montero et al., 2017) to interrupt the positive feedback established between senescence and tumor plasticity events. However, both SASP molecules and these signaling pathways play critical physiological roles too, so acting on them can be complex and lead to significant side effects. Thus, the use of therapies targeting SnCs and their secretome might be rationally defined depending on the tumor type, the primary treatment chosen, and the tissue context where that tumor developed. All these aspects make SnCs a central player that must be better understood in the context of carcinogenesis and response to therapy, having potential as a marker for association with prognosis and targeted therapeutic modulation.
